# Research on Determining Elastic–Plastic Constitutive Parameters of Materials from Load Depth Curves Based on Nanoindentation Technology

**DOI:** 10.3390/mi14051051

**Published:** 2023-05-15

**Authors:** Zhentao Li, Yun Ye, Guanjun Zhang, Fengjiao Guan, Junjie Luo, Panfeng Wang, Jiao Zhao, Li Zhao

**Affiliations:** 1 State Key Laboratory of Advanced Design and Manufacturing for Vehicle Body, Hunan University, Changsha 410082, China; 2 Institute of New Materials, Guangdong Academy of Sciences, National Engineering Laboratory of Modern Materials Surface Engineering Technology, Guangdong Provincial Key Laboratory of Modern Surface Engineering Technology, Guangzhou 510651, China; 3 Laboratory of Science and Technology on Integrated Logistics Support, College of Intelligence Science and Technology, National University of Defense Technology, Changsha 410073, China

**Keywords:** nanoindentation, inverse estimation, elastoplastic property, spherical indentation curves, cyclic loading

## Abstract

It is of great significance for structural design and engineering evaluation to obtain the elastic–plastic parameters of materials. The inverse estimation of elastic–plastic parameters of materials based on nanoindentation technology has been applied in many pieces of research, but it has proved to be difficult to determine the elastic–plastic properties of materials by only using a single indentation curve. A new optimal inversion strategy based on a spherical indentation curve was proposed to obtain the elastoplastic parameters (the Young’s modulus *E*, yield strength *σ_y_*, and hardening exponent *n*) of materials in this study. A high-precision finite element model of indentation with a spherical indenter (radius R = 20 µm) was established, and the relationship between the three parameters and indentation response was analyzed using the design of experiment (DOE) method. The well-posed problem of inverse estimation under different maximum indentation depths (*h_max_*_1_ = 0.06 R, *h_max_*_2_ = 0.1 R, *h_max_*_3_ = 0.2 R, *h_max_*_4_ = 0.3 R) was explored based on numerical simulations. The results show that the unique solution with high accuracy can be obtained under different maximum press-in depths (the minimum error was within 0.2% and the maximum error was up to 1.5%). Next, the load-depth curves of Q355 were obtained by a cyclic loading nanoindentation experiment, and the elastic–plastic parameters of Q355 were determined by the proposed inverse-estimation strategy based on the average indentation load-depth curve. The results showed that the optimized load-depth curve was in good agreement with the experimental curve, and the optimized stress–strain curve was slightly different from the tensile test, and the obtained parameters were basically consistent with the existing research.

## 1. Introduction

Characterization of the elastic–plastic properties of materials is of great significance for the structural design and engineering evaluation. Due to the incomparable advantages in the measurement of nondestructive and micro-mechanical properties [1,2], nanoindentation technology has been widely used in the research of mechanical properties of metals [3,4,5,6], films [7,8,9], and biomaterials [10,11,12], particularly in the elastic–plastic properties.

For several decades, many researchers have focused on extracting the Young’s modulus (*E*), yield strength (*σ_y_*), and hardening exponent (*n*) of elastic–plastic materials from indentation load-depth curves. Based on the method of Oliver and Pharr [13,14] or Hertzian contact theory [15], Young’s modulus can be determined from the indentation curve. However, the material parameters obtained from these experiments depend heavily on the contact radius [16,17], which plays an important role in the effects of the accuracy of measurement of parameters. Although many scholars have revised the calibration of contact function which plays an important role in the calculation of the tip area function, it is still difficult to obtain a high-precision area radius [18,19]. Moreover, the parameters directly obtained such as the indentation modulus and hardness are not enough to characterize the elastic–plastic properties of materials.

The difficulty in obtaining the elastic–plastic parameters of materials from the load-depth curve is that the relationship between them is not one-to-one, which means the combination of different material parameters will produce the same load-depth curve [20,21,22,23,24]. Based on the reverse method, some scholars have used dual indenters to distinguish the load-depth curves of different material combinations for determining the unique solution of elastic–plastic parameters [25,26,27,28]. However, it is difficult to find a suitable indenter to distinguish the indentation curves of different material parameter combinations, especially for some unknown materials [29]. In addition, the number of solving parameters corresponds to the number of indenters, and a large number of coefficients need to be fitted by finite element calculation in practical application. The representative strain proposed by Tabor [30] has been applied to solve the elastic–plastic parameters of different materials [31,32,33,34], which effectively reduces the fitting coefficient. The key of this method is to determine the appropriate representative strain; however, unfortunately, the definition of this is still controversial [32,34,35,36].

The inverse estimation approach of elastoplastic parameters is also often used in research. Ensuring the uniqueness of the material parameter is the main problem in the inverse estimation process. By introducing an additional indentation parameter, pile-up/sink-in, some scholars [22,37,38] have obtained the unique solution of elastic–plastic properties of materials combined with the load-depth curve. However, accurately measuring the morphology of residual indentation is difficult and costly. Moreover, there is no pile-up/sink-in observed in the indentation process for some materials [39,40,41]. A spherical indenter can separate the elasticity and plasticity of materials [42], and it had been always used to solve multi-solution problems [42,43,44]. In recent years, cyclic loading with a spherical indenter has been gradually applied to obtain the solution of elastic–plastic properties of materials [3,4,45,46,47,48,49]. As far as this is concerned, only a few researchers have carried out the reverse engineering of elastic–plastic properties of materials based on the depth curve of cyclic loading [49], and the uniqueness of solutions has not been explained in their research. In addition, it is still unknown what kind of influence the coupling relationship between the elastic–plastic parameters of various materials has on the cyclic loading–unloading curve.

The purpose of this study is to acquire the elastic–plastic constitutive parameters of materials from the load-depth curve obtained based on nanoindentation indentation technology. The uniqueness of the solution at different indentation depths was discussed in detail using finite element simulation. Combined with the design of experiment (DOE) method, the relationship between elastic–plastic parameters and indentation curve was further studied. Finally, the proposed inverse estimation strategy was used to determine the elastic–plastic parameters of steel Q355 based on the indentation experiment load-depth curve, and the results were compared with those of the tensile test.

## 2. Identification Procedure

In this study, a metamodel-based global optimization strategy has been implemented in LSOPT. By parameterizing the material parameters in ABAQUS calculation input files, the material parameters were modified and new input files were generated. The ABAQUS run for calculation was based on a batch file by using the user-defined interface, and the python script was used to extract the load-depth curves from the resulting ABAQUS output files which were successfully calculated. The genetic algorithm (GA) provided by LSOPT was widely used in engineering optimization because of its powerful global optimal search ability [50,51]. The Kriging metamodel was used to replace the complicated finite element calculation in optimization to improve the calculation efficiency [52,53].

In this study, a power law strain-hardening curve was used to simulate the indentation experiments. The stress–strain relationship can be described as:

(1) $$\left\{\begin{array}{c} \sigma =E\varepsilon ;\left(\sigma \leq \sigma_{y}\right)\\ \sigma =K\varepsilon^{n},\left(\sigma \geq \sigma_{y}\right) \end{array}\right.$$

where *E* is the Young’s modulus; *σ_y_* the yields tress; *K* the strength coefficient; *n* the strain hardening component. By enforcing continuity at *σ* = *σ_y_*,

(2) $$\sigma_{y}=E\varepsilon_{y}=K\varepsilon_{y}^{n}$$

The decomposition of the strain into elastic and plastic parts is given by:

(3) $$\varepsilon =\varepsilon_{y}+\varepsilon_{p}$$

Then, the stress equation can be written as:

(4) $$\sigma =\sigma_{y}{\left(1+\frac{E}{\sigma_{y}}\varepsilon_{p}\right)}^{n}$$

*E*, *n,* and *σ_y_*, are used to define the elastic–plastic relationship of materials in ABAQUS simulation by Equation (4).

**Figure 1:**
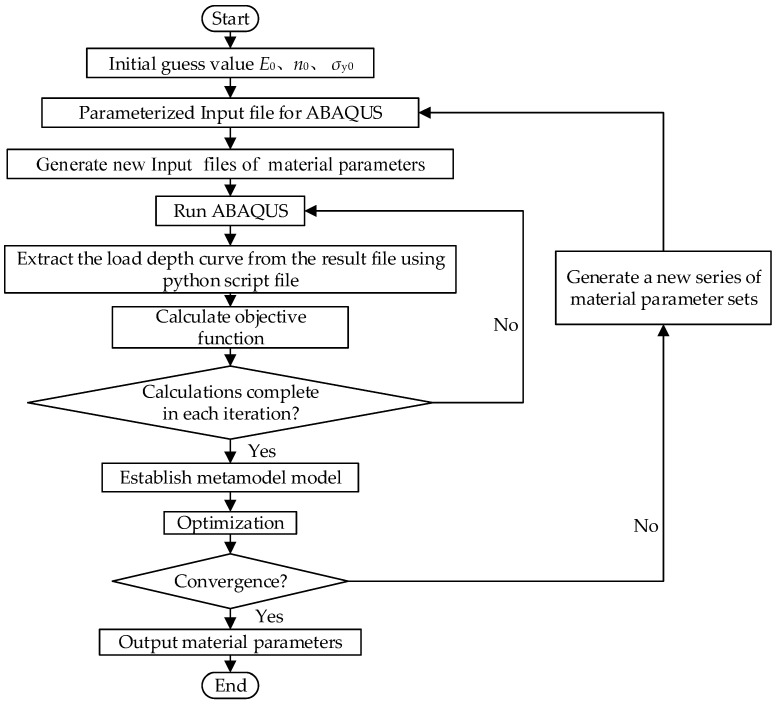
Flow chart of the optimization to determine the properties from the load-depth curves.

The procedure (Figure 1) can be described in the following steps: firstly, the load-depth curves of multiple cyclic loading are obtained by nanoindentation experiment; secondly, a high-precision model of indentation simulation was established by finite element analysis; next, a set of conjectured initial values (*E*_0_, *n*_0_, *σ_y_*_0_) were substituted into the finite element model for calculation, and the error value of the area between the simulation curve and the experimental curve was defined as the objective function, which could be automatically interpolated in LSOPT by Equation (5):

(5) $$F\left(E,n,\sigma_{y}\right)=\int_{h_{1}}^{h_{2}}\left|P^{sim}\left(h\right)-P^{exp}(h)\right|$$

In the process of iteration, the design variables were constantly changing in the set domain of interest to complete the new calculation. After finishing an iteration, a Kriging metamodel was established according to a series of material parameters and corresponding objective function responses, and the optimal solution in this iteration was predicted based on the metamodel so that the indentation simulation curve can better match the experimental curve. The optimization model can be described as:

(6) $$\min F(E,n,\sigma_{y})$$

When the convergence condition reached the objective function tolerance or maximum number of iterations, the optimization process would be completed, and the material parameters would be determined with the simulation curve consistent with the experimental curve.

## 3. Numerical Study

### 3.1. Finite Element Simulation

**Figure 2:**
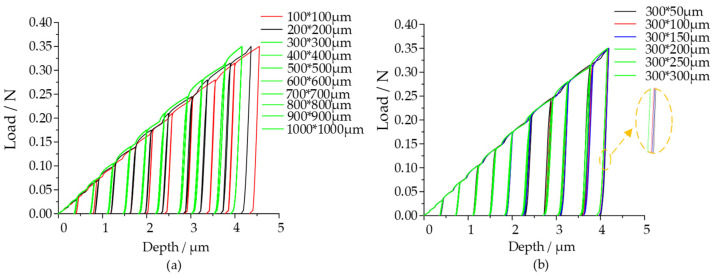
Model dimension size sensitivity analysis. (**a**) length and width; (**b**) width.

The commercial finite element software ABAQUS was used to establish an axisymmetric two-dimensional model to simulate the indentation test of spherical indenter [49]. In order to avoid the size effect [54], the sensitivity analysis of the model size (simulated volume) was carried out. When the length and width of the model were less than 10 times and 5 times of the radius (R) of the indenter, respectively, the size effect was obvious (Figure 2) which was consistent with the conclusion obtained by Huang et al. (2019) [49]. Finally, the indentation model size was 150 μm × 100 μm, it should be noted that the same color (green) in Figure 2 means that they are perfectly superimposed.

**Figure 3:**
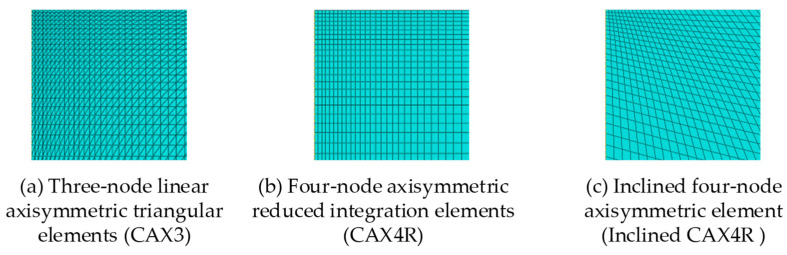
Schematic diagram of three types of elements.

**Figure 4:**
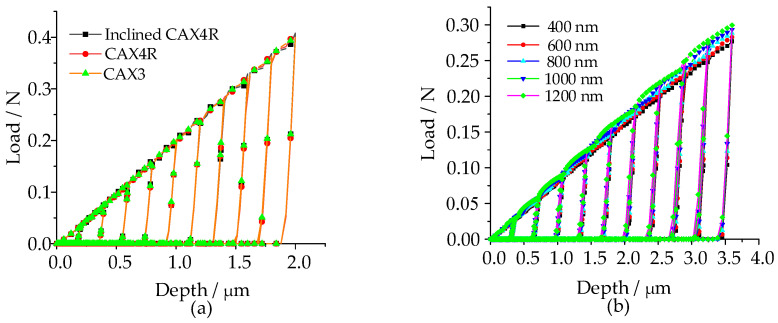
Mesh sensitivity analysis. (**a**) element types; (**b**) element size.

**Figure 5:**
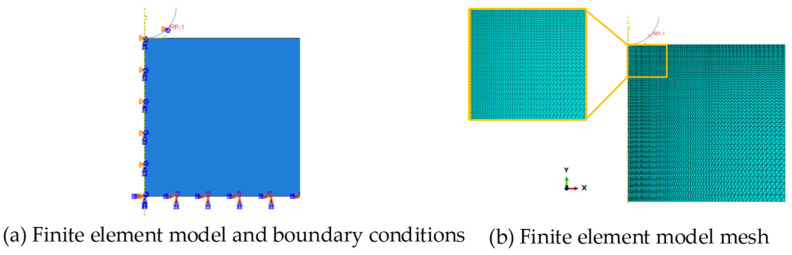
Indentation simulation model.

The spherical indenter was defined as an analytical rigid surface with a radius of 20 µm, and three types of elements (Figure 3) [27,37,55], and the size of the element was discussed in detail. The results of the mesh sensitivity analysis showed (Figure 4a) that the load-depth curves obtained by three commonly used mesh types were consistent with each other, among which the 3-node asymmetric linear triangular elements (CAX3) had a fast convergence speed. Hence, asymmetric linear triangular elements of type CAX3 were used to divide samples. Element accuracy not only affects the convergence result, but also affects the load depth curve (Figure 4b). The area contacted with the indenter was divided into finer meshes while other areas were divided into coarser meshes (Figure 5, minimum mesh size: 400 nm, maximum mesh size: 1.2 µm, total mesh number: 11,368, node number: 5841).

A reference point was defined on the indenter, where the displacement loading of 10% (0.1 µm) of the maximum indentation depth in each analysis step was applied. The contact between the indenter and the sample surface was set to face-to-face contact, the friction coefficient was set to 0.1 to reduce the influence [56], and the Poisson’s ratio was fixed to 0.3 [45,49].

### 3.2. DOE Analysis

DOE analysis was carried out in commercial software ISIGHT to study the influence of material parameters on indentation response. Three material parameters were used as the input variables of the design, and the difference between the “experimental” load-depth curve and the simulated load experiment curve was taken as the response. Latin hypercube design method was used to uniformly sample the design space to evaluate the main effect and interaction effect between material parameters (the Young’s modulus spanning from 100 GPa to 300 GPa, the yield stress spanning from 100 MPa to 550 MPa, the hardening exponent spanning from 0.1 to 0.6). A total of 50 groups of material combinations were evenly obtained in the design space, and 0.1% correction was adopted to replace the wrong points in the middle calculation to recalculate to ensure the number of samples (for more details please see Table A1 in Appendix A).

**Figure 6:**
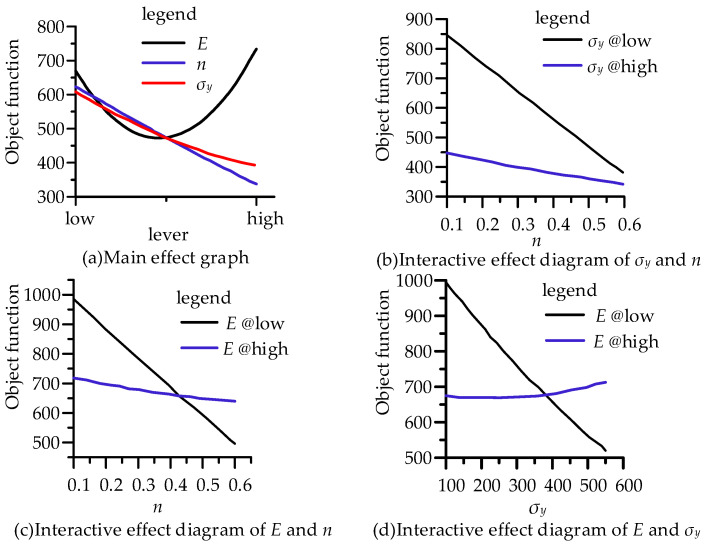
Main effect graph and interactive effect diagram.

**Figure 7:**
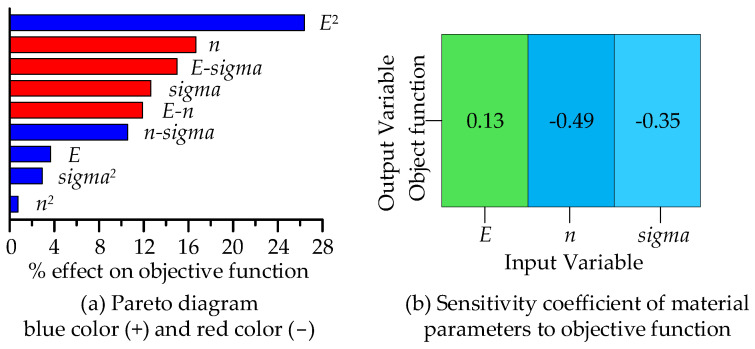
Pareto diagram (**a**) and global sensitivity analysis (**b**).

The hardening component and yield stress had a linear negative main effect on the objective function, while Young’s modulus had a second-order main effect on the objective function (Figure 6a). The interaction among the three parameter factors was significant (Figure 6). The interaction between Young’s modulus and the yield stress had the greatest influence on the objective function, while the interaction between the yield stress and the hardening component had the smallest influence. These were also reflected in the Pareto analysis diagram (Figure 7a) by the contribution degree of the main effect and interaction effect to the objective function, in which the blue bar represents the positive effect and the red represents the negative effect. Figure 7a describes the influence of Young’s modulus, yield stress, hardening component, and factor interaction on the response in detail, and Figure 7b gives the global sensitivity coefficients of the three variables to the response, which can specifically show the sensitivity of Young’s modulus, the yield stress, and the hardening index to the response results. Among them, Young’s modulus had a positive effect on the objective function (Figure 7b), but the influence was the smallest (13%). The yield stress and the hardening component had negative effects on the objective function, and the influence of the hardening component (49%) was greater than that of the yield stress (35%).

### 3.3. Study of the Uniqueness of the Solution

Most researchers have used the maximum indentation depth spanning from 0.1 R to 0.3 R to study the elastic–plastic properties of materials. In fact, the indentation depth of a spherical indenter at 0.2 R can effectively identify the plastic characteristics of materials [56]. However, in the shallow indentation test of a spherical indenter (*h_max_* ≤ 0.06 R), different materials would produce the same load-depth curve [57], which means that the different indentation depths may also bring multi-solution problems. Hence, the uniqueness of the solution of mechanical property parameters with the inverse solution strategy was analyzed under different maximum indentation depths.

The material parameters of Al5083 in the literature (*E*: 80 GPa, *σ_y_*: 140 MPa, *n*: 0.21) [58] were given to the finite element model, and four different maximum indentation depths were applied, respectively: *h_max_*_1_ = 1200 nm (0.06 R), *h_max_*_2_ = 2000 nm (0.1 R), *h_max_*_3_ = 4000 nm (0.2 R), *h_max_*_4_ = 6000 nm (0.3 R). Finally, four load-depth curves of cyclic loading at different maximum indentation depths were obtained, which were taken as the test target curves. Three groups of different initial values (*E*_0_, *n*_0_, *σ_y_*_0_) in the same domain of interest were set to identify the material parameters (Table 1) and the optimized results were compared with the experimental values to study the uniqueness of the solution.

**Table 1:** Initial value and domain of interest.

	*E*_0_ (GPa)	*n*_0_	*σ_y_*_0_ (MPa)
T1	50	0.2	80
T2	90	0.5	120
T3	120	0.8	200
Boundaries	20 < *E* < 150	0.01 < *n* < 1	30 < *σ_y_* < 600

The optimization results are summarized in Table 2. In the optimization process, three parameters were changed simultaneously to obtain the optimal solution to match the target curve. Various ranges of initial values were used to exploring the uniqueness of solutions. In the solution results of three maximum indentation depths (0.06 R, 0.1 R, 0.2 R), the three material parameters optimized with different initial values were basically consistent with the real target values, and the relative error was less than 0.2%. When the maximum indentation depth increased to 6 µm (0.3 R), only the T1 group could finish the optimization normally, and the other two groups could not finish the optimization to the non-convergence of finite element calculation in the iterations, and the final parameter optimization error was less than 1.5%. Compared with other indentation depths, the error of optimized values at this indentation depth increased. With the increase in the maximum indentation depth, the finite element model needed a more precise mesh to achieve convergence; otherwise, the finite element mesh with a high degree of dispersion will have a larger error on the load-depth curve, which will lead to an increase in the error of the parameters.

**Figure 8:**
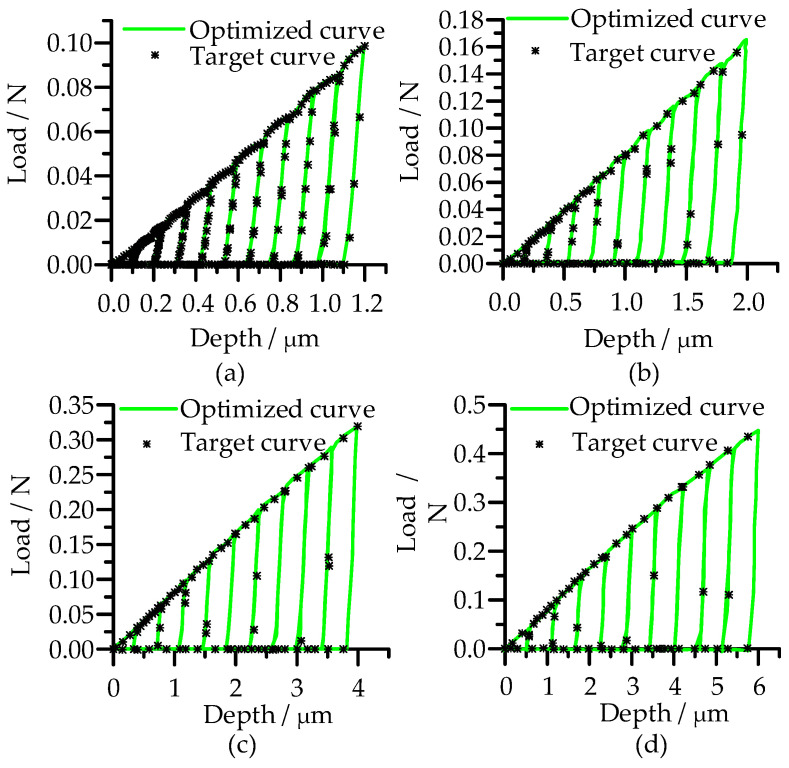
Target load-depth curves and optimized curves under different maximum indentation depth: (**a**) *h*_max1_: 1.2 µm; (**b**) *h*_max2_: 2 µm; (**c**) *h*_max3_: 4 µm; (**d**) *h*_max4_: 6 µm.

**Figure 9:**
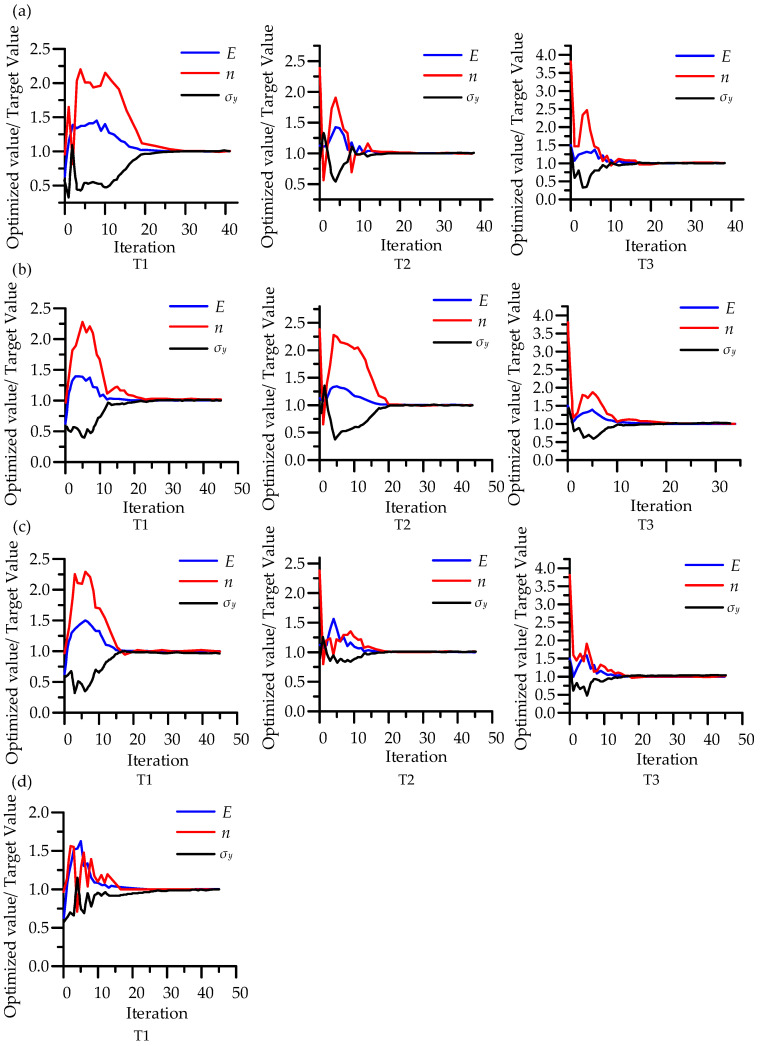
Optimized parameter values versus iterations for different maximum indentation depths (**a**) *h_max_*_1_: 1.2 µm; (**b**) *h_max_*_2_: 2 µm; (**c**) *h_max_*_3_: 4 µm; (**d**) *h_max_*_4_: 6 µm.

When different initial values were used, the number of parameter iterations would be slightly different, and the closer the initial value was to the target value, the less the number of iterations would be in optimization. In any case, the optimized load-depth curves at different maximum indentation depths all matched well with the target curves (Figure 8). With the increase in indentation depth, the number of iterations of parameter optimization convergence increase, but they all converge on the target value at last (Figure 9).

## 4. Experimental Validation

### 4.1. Sample Preparation

Q355 steel was used for the experiment to verify the optimization method. The steel plate was processed into a 10 mm × 10 mm × 3 mm nanoindentation prefabricated sample by wire cutting, which was enough to ensure the spacing of indentation points (the spacing distance was 100 µm). The tensile specimens were obtained using wire cutting from the same plate. The samples were processed in three directions to eliminate the influence of material anisotropy. After the prefabricated sample was cleaned by ultrasonic wave, the nanoindentation sample was obtained by thermal inlay with epoxy resin. The sample was ground with P80, P1000, and P2000 sandpaper in turn, and then the surface was polished to be smooth and scratch-free using a 3 µm, 1 µm, and 0.05 µm polishing flannel with the corresponding suspension.

### 4.2. Mechanical Test

The micro-nanoindentation test was carried out by using a NHT3 indenter (Anton Paar, Switzerland and Austria) and a diamond spherical indenter with a radius of 20 μm, the maximum indentation load of which can reach up to 500 mN. Considering the load range and proper indentation depth of the instrument, the maximum indentation depth of Q355 was 2 µm (0.1 R). A cyclic loading experiment was carried out on a nanoindentation specimen, and six load-depth curves of the Q355 indentation test were obtained. In the experiment, the displacement loading mode was adopted. The experiment was carried out by loading and unloading at a constant rate of 1.2 µm/min to the maximum indentation depth. The sampling frequency was set to 100 Hz to record the time history of load and depth, and finally the load-depth curve was output.

**Figure 10:**
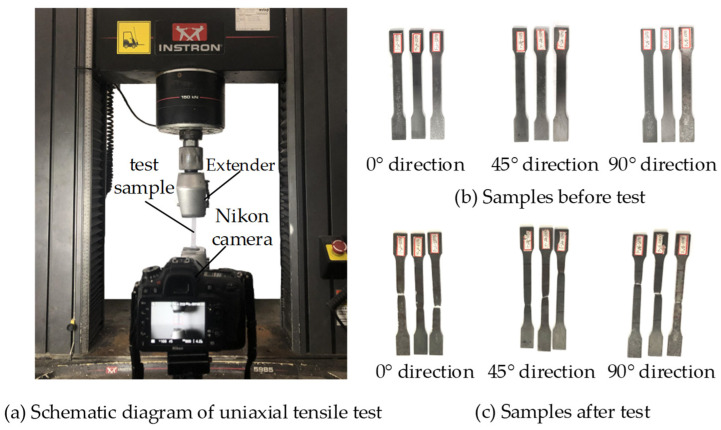
Schematic diagram of tensile test.

The INSTRON5985 tensile testing machine and VIC-2D (CSI, USA) strain measurement system were used for the tensile test (Figure 10). Before the test, the VIC system was calibrated to ensure that the Nikon D7100 camera had high accuracy (the deviation was less than 0.3 pixels and the size deviation was less than 0.03 mm). The sampling frequency of the camera was set to 1 frame/4 s. The test was carried out at room temperature (temperature 25 °C, humidity 33%), and the displacement loading control mode was adopted. The loading rate was 2 mm/min, and the strain rate was within 0.01/s until the specimen was pulled off. The samples before and after the experiment are showed as Figure 10.

### 4.3. Comparations and Results

**Figure 11:**
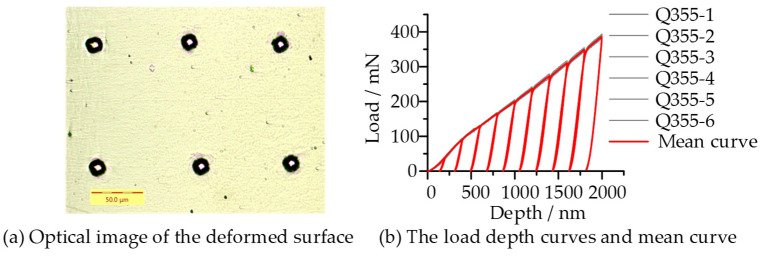
Results of the indentation experiment.

**Figure 12:**
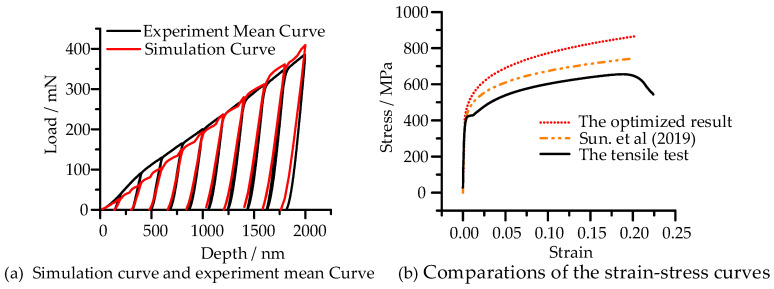
Optimized results of indentation test curve based on Q355 [4].

Figure 11a shows the residual morphology characteristics of six spherical indentation tests, and the load-depth curves of six indentation tests had high consistency and repeatability (Figure 11b). The mean curve of six load-depth curves was obtained through normalization solution, which was used as the target curve for solution (Figure 11b) The initial values and the lower and upper boundaries of the design variables in the optimization process and the optimized value are shown in Table 3. From the results of the benchmark curve (Figure 12a), the simulation curve basically matches the experimental curve well into the loading stage. However, in the unloading section, the simulation curve was steeper than the experimental curve, which reflected that the elastic modulus calculated by simulation was slightly smaller than the value measured by experiment. The hardening exponent was close to the result of Sun et al. (*n* = 0.142) (2019) [4] and the yield stress was larger than their research result (*σ_y_* = 381 MPa), which was basically consistent with the results obtained by Li et al. (2020) [59] by cyclic loading indentation test with a spherical indenter (R = 500 µm, *σ_y_* = 424 MPa) and slightly larger than the yield stress obtained by the compression test (*σ_y_* = 392 MPa). From the comparations of the stress–strain curve in Figure 12b, it shows that the stress–strain curve of tensile experiment was lower than that of the indentation experiment in the study of Sun et al. (2019), and the stress–strain curve obtained in this study was higher than both of them [4].

## 5. Discussion

In this study, the feasibility of the proposed optimization reverse algorithm was verified by numerical simulation and experiment. In the solution of numerical simulation, the optimized load-depth curve under each maximum indentation depth was in good agreement with the “simulation” test curve, and the solution relative error of the three material parameters was controlled within 0.01%, which was much higher than the parameter accuracy obtained by Collin et al. (2008) using the reverse method based on the spherical indentation load curve (the relative error is 9% on *σ_y_*, 21% on *K* and 3% on *m*) [45]. However, when applied to the real test load curve, although the real test load curve basically coincided with the curve calculated by optimization, and the obtained parameters were basically consistent with those in the literature, we found that the stress–strain curve fitted by optimization was obviously different from that obtained by tensile test, which may be caused by different test methods.

**Figure A1:**
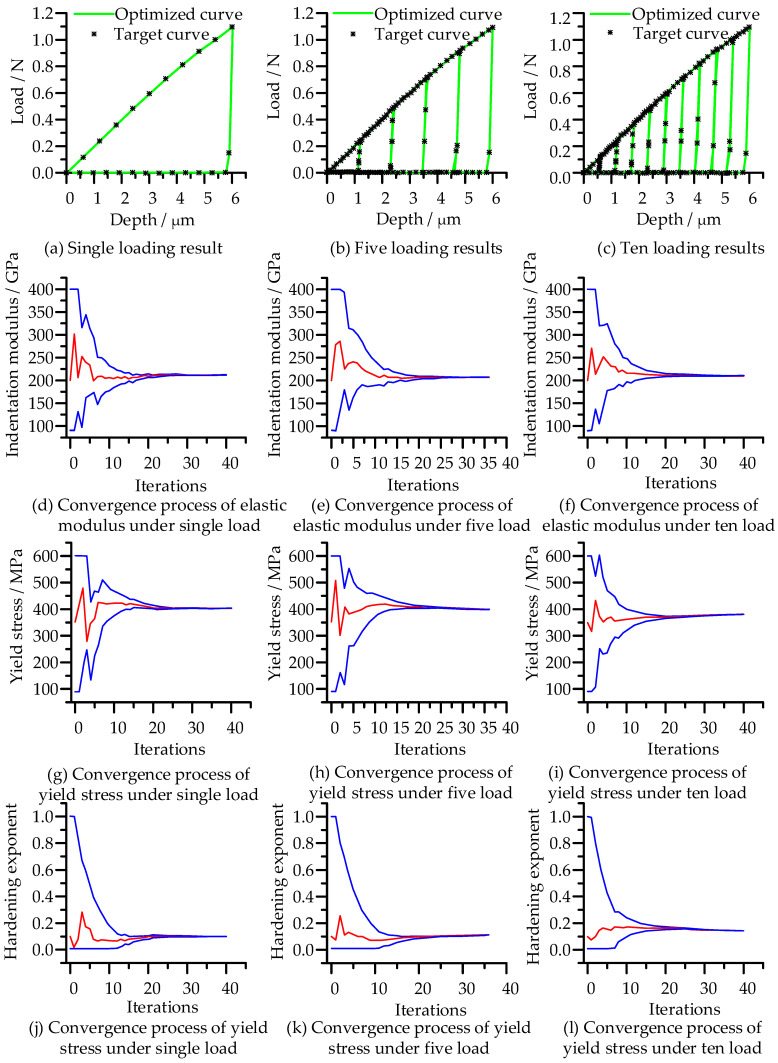
Parameter iteration history and curve matching results of different cyclic loading times for exploring the influence of loading times on parameter inverse estimation in Section 5, Discussions.

The difference between simulated load-depth curve and experimental curve may affect the result of the identification algorithm. There is a difference between the real cyclic loading–unloading curve and the simulation test curve; in particular, in the unloading section, the real pressure-head shape may affect the unloading curve, which could influence the Young’s modulus [45]. In addition, the effect of cyclic loading strengthens the hardening characteristics of the material, which can effectively reflect the plasticity and hardening characteristics of the material. Based on the parameters in the literature (*E*: 210 GPa, *n*: 0.14, *σ_y_*: 381 MPa), the finite element simulation of steel was carried out to study the influence of the reduction in cyclic loading times on parameter identification (for more details please see Table A2 and Figure A1 in Appendix B). The results showed that the higher the number of cyclic loading and unloading, the higher the accuracy of the sigma and hardening exponent; however, with the increase in cyclic loading and unloading times, the error of Young’s modulus may be accumulated and superimposed.

The sensitivity of material parameters to objective function may also affect the identification results. In the DOE analysis, the sensitivity of the elastic modulus to objective function was the lowest (negative effect), and the interactions between the elastic modulus and hardening exponent, Young’s modulus, and yield stress were the most obvious (positive effect). The relationship among the three parameters was complex and highly coupled, which made it difficult to judge how much the elastic modulus was helpful in solving the objective function, which may cause the influence of the elastic modulus to be offset when solving the objective function based on the experimental curve.

## 6. Conclusions

In this study, a new inverse method of elastic–plastic parameters based on the load curve of a spherical indenter was proposed. The results of the numerical analysis showed that the proposed recognition algorithm could obtain high-precision solutions (0.2%) at different pressing depths. At the same time, the results of the DOE analysis showed that there was a high coupling relationship among the three parameters. The elastic modulus has the least influence on the objective function with a second-order nonlinear relationship. In the reverse verification of our experiment, the elastic modulus is smaller than that of the tensile test, but the obtained three parameters are still acceptable compared with the literature, which indicates that the results based on the proposed algorithm are reliable.

## Figures and Tables

**Table 2:** Optimization results of three parameters with different maximum indentation depths.

*h_max_*	Test	*E* (GPa)	Error (%)	*n*	Error (%)	*σ_y_* (MPa)	Error (%)
1200 nm	T1	80.0010	0.00125	0.210026	0.01238	139.99400	0.00429
	T2	80.0009	0.00112	0.210022	0.01048	139.99500	0.00357
	T3	80.0001	0.00013	0.209998	0.00095	140.00100	0.00071
2000 nm	T1	80.0074	0.00925	0.210284	0.13524	139.93700	0.04500
	T2	79.9991	0.00112	0.209971	0.01381	140.00700	0.00500
	T3	79.9990	0.00125	0.209993	0.00333	140.00200	0.00143
4000 nm	T1	79.9836	0.02050	0.209773	0.10810	140.05800	0.04143
	T2	79.9942	0.00725	0.209912	0.04190	140.02200	0.01571
	T3	79.9821	0.02237	0.209741	0.12333	140.06600	0.04714
6000 nm	T1	80.1152	0.14400	0.212645	1.25952	139.36300	0.45500

**Table 3:** Initial values and the lower and upper boundaries of the design variables in the optimization process and the optimized value.

Variable Name	Initial Value	Lower Bound	Upper Bound	Optimized Value
*E* (GPa)	210	189	231	189.000
*n*	0.2	0.1	0.3	0.162
*σ_y_* (GPa)	350	300	500	416.071

## Data Availability

Data available on request due to restrictions eg privacy or ethical. The data presented in this study are available on request from the corresponding author. The data are not publicly available due to confidentiality of institutional data.

## Appendix A. Latin Hypercube Design Matrix of Material Parameters

**Table A1:** Latin hypercube sampling design matrix of three material parameters used for DOE analysis in Section 3.2, DOE analysis.

Number	*E* (MPa)	*n*	*σ_y_* (MPa)	Number	*E* (MPa)	*n*	*σ_y_* (MPa)
1	294,081.633	0.294	292.860	26	146,122.449	0.518	155.100
2	199,387.755	0.437	467.350	27	163,877.551	0.304	504.080
3	234,897.959	0.386	338.780	28	21,836.735	0.447	366.330
4	175,714.286	0.508	338.780	29	98,775.510	0.457	274.490
5	205,306.122	0.222	100.000	30	57,346.939	0.549	145.920
6	81,020.408	0.355	550.000	31	157,959.184	0.171	320.410
7	63,265.306	0.314	302.040	32	104,693.878	0.243	412.240
8	217,142.857	0.253	403.060	33	10,000.000	0.202	357.140
9	122,448.980	0.600	265.310	34	163,877.551	0.365	109.180
10	193,469.388	0.416	228.570	35	92,857.143	0.559	393.880
11	252,653.061	0.141	311.220	36	69,183.673	0.151	246.940
12	128,367.347	0.284	201.020	37	33,673.469	0.569	283.670
13	45,510.204	0.488	494.900	38	110,612.245	0.161	118.370
14	187,551.020	0.590	458.160	39	264,489.796	0.529	522.450
15	15,918.367	0.427	210.200	40	181,632.653	0.120	191.840
16	288,163.265	0.467	256.120	41	223,061.224	0.580	219.390
17	58,571.429	0.539	375.510	42	39,591.837	0.273	173.470
18	27,755.102	0.324	439.800	43	51,428.571	0.192	513.270
19	228,979.592	0.110	448.980	44	75,102.041	0.100	384.690
20	270,408.163	0.345	164.290	45	276,326.531	0.182	182.650
21	86,938.776	0.396	136.730	46	211,224.490	0.263	237.760
22	134,285.714	0.498	540.820	47	282,244.898	0.365	476.530
23	116,530.612	0.406	430.610	48	300,000.000	0.212	421.430
24	246,734.694	0.233	531.630	49	140,204.082	0.131	485.710
25	152,040.816	0.335	329.590	50	240,816.327	0.478	127.550

## Appendix B. Inversion Results of Parameters with Different Loading Times

**Table A2:** Results of optimization strategy under different loading times in Section 5, Discussions.

Loading Times	*E* (GPa)	Error (%)	*n*	Error (%)	*σ_y_* (MPa)	Error (%)
1	211.547	0.737	0.100	28.618	403.518	5.910
5	207.215	1.326	0.111	20.435	398.457	4.582
10	210.089	0.042	0.143	2.235	380.354	0.170
